# Three-dimensional quantitative analysis of dental and skeletal characteristics of skeletal Class I unilateral posterior crossbite in adults

**DOI:** 10.1186/s12903-022-02622-3

**Published:** 2022-12-10

**Authors:** Jingwen Wu, Joanna Ser Yun Bek, Mengqi Luo, Hao Xu, Yanmin Wang, Xianglong Han

**Affiliations:** 1 State Key Laboratory of Oral Diseases, National Clinical Research Center for Oral Diseases, Department of Orthodontics, West China Hospital of Stomatology, Sichuan University, Chengdu, 610041 Sichuan People’s Republic of China; 2 State Key Laboratory of Oral Diseases, National Clinical Research Center for Oral Diseases, Research Unit of Oral Carcinogenesis and Management, Chinese Academy of Medical Sciences, West China Hospital of Stomatology, Sichuan University, Chengdu, 610041 Sichuan People’s Republic of China

**Keywords:** Dentoskeletal characteristics, Facial asymmetry, Transverse discrepancy, Unilateral posterior crossbite

## Abstract

**Background:**

To study the dentoskeletal characteristics and the degree of compensations in skeletal Class I adults with unilateral posterior crossbite (UPCB).

**Methods:**

A sample of 40 adults was chosen for this cross-sectional study. 20 skeletal Class I adults with UPCB (mean age: 22.20 ± 2.88 years), were compared to 20 skeletal Class I adults with normal occlusion (mean age: 27.56 ± 5.76 years). The respective dentoskeletal measurements were made on cross-sectional images from cone-beam computed tomography scans.

**Results:**

Skeletally, both groups showed significant differences (*P* < 0.05) in mandibular corpus length and menton deviation with the UPCB group showing the greatest displacement. Maxillomandibular vertical asymmetry and condylar positional asymmetry were not significant in both groups (*P* > 0.05). For dental variables on the second premolar and first molar, the UPCB group showed greater linear and angular differences when compared to the control group (*P* < 0.05). On the crossbite side, maxillary posterior teeth were more buccally inclined, and mandibular posterior teeth were more lingually inclined. However, on the non-crossbite side, both maxillary and mandibular posterior teeth were lingually inclined.

**Conclusion:**

Adults with UPCB showed distinct transverse dentoskeletal asymmetry. No asymmetry was found in the condylar position and the mandibular height in UPCB adults.

## Background

Posterior crossbite has been defined as a transverse arch discrepancy in which the palatal cusps of one or more upper posterior teeth do not occlude in the central fossae of the opposing lower teeth [1]. The prevalence of posterior crossbite reported ranges from 7.7 to 22% in adolescents, with a predominance of unilateral posterior crossbite (UPCB) [2, 3]. Among adults, the incidence of UPCB is even higher, which is between 10 and 30% [4].

Untreated UPCB might be an etiologic factor of progressive anatomical skeletal asymmetry [2, 5, 6]. Approximately 79–90% of children with UPCB presented with a mandibular functional shift [2, 3], but this is hardly detected in UPCB adults. Some studies [7, 8] demonstrated that this phenomenon can be explained by the progressive asymmetric adaptation that gradually leads to permanent structural asymmetry. Compared with early correction, treatment of UPCB in adults is relatively more complex since the expansion of the dental arch is limited, and the underlying skeletal asymmetries might be neglected. It must be decided whether the posterior crossbite is a true skeletal asymmetry or only involves dentoalveolar structures before determining a treatment plan.

However, the extent to which UPCB influences dentoalveolar and skeletal structures and their relationships remain controversial due to study design variations, sample grouping, and radiographic assessment methods [7, 9–12]. Previous studies that involved UPCB adults have mainly focused on the transverse dentoskeletal asymmetries and restricted to Caucasians demographically [7, 9]. Nevertheless, the transverse dentoskeletal morphology was reported to be affected by both sagittal and vertical skeletal discrepancies as well [13, 14]. Furthermore, most of the assessments were computed on two-dimensional (2D) radiographs with inherent limitations such as unwanted magnification and superimposition. Compared with 2D radiographs, cone-beam computed tomography (CBCT) could provide three-dimensional (3D) assessments and substantially reduce the distortion of the images [15, 16].

Although the assessment of mandibular and dental arch asymmetry in UPCB patients has been of great interest in previous literature [7, 9, 11, 12], few studies [7] have systematically studied both dentoalveolar and skeletal adaption patterns in UPCB adults. Therefore, the present study aims to study the dentoskeletal characteristics and the degree of compensation in skeletal Class I adults with UPCB when compared to the adults with skeletal Class I, normal occlusion using CBCT. The tested null hypothesis is that skeletal Class I UPCB adults do not exhibit a significantly greater degree of maxillomandibular skeletal asymmetry between the crossbite and non-crossbite side compared to adults with skeletal Class I normal occlusion.

## Methods

The sample of this study consists of 40 adults, referrals from the West China School of Stomatology, Sichuan University, Chengdu, China. The study was approved by the Institutional Review Board of West China School of Stomatology (approval number WCHSIRB-D-2018-131). 20 normal occlusion skeletal Class I adults (7 males, 13 females; 27.56 ± 5.76 years old) were selected for the control group, and the UPCB group comprised 20 skeletal Class I adults (8 males, 12 females; 22.20 ± 2.88 years old) with UPCB, the enrollment criteria are shown in Table 1.

**Table 1:** Inclusion criteria for sample selection

Control group	UPCB group
Dental and skeletal Class I (0° ≤ ANB < 4°) [17] relationship without UPCB	Skeletal Class I (0° ≤ ANB < 4°) [17] malocclusion with UPCB involving at least two posterior teeth, Edge-to-edge tooth was excluded
Average growth pattern (20° ≤ FMA ≤ 30°) [18]	Average growth pattern (20° ≤ FMA ≤ 30°) [18]
All teeth present except the third molars	All teeth present except the third molars
No or mild crowding (< 4 mm)	No or mild crowding (< 4 mm)
Condyle and glenoid fossa with continuous cortical bone	Condyle and glenoid fossa with continuous cortical bone
No previous orthodontic treatment or craniofacial surgery	No previous orthodontic treatment or craniofacial surgery
No crown or cuspal restorations, and no or mild tooth abrasion	No crown or cuspal restorations, and no or mild tooth abrasion
Absence of congenital malformations and craniofacial anomalies	Absence of congenital malformations and craniofacial anomalies

*ANB* Angle formed by A-point-nasion-B-point; *FMA* the angle between FH plane and mandibular plane; *UPCB* unilateral posterior crossbite

**Fig. 1:**
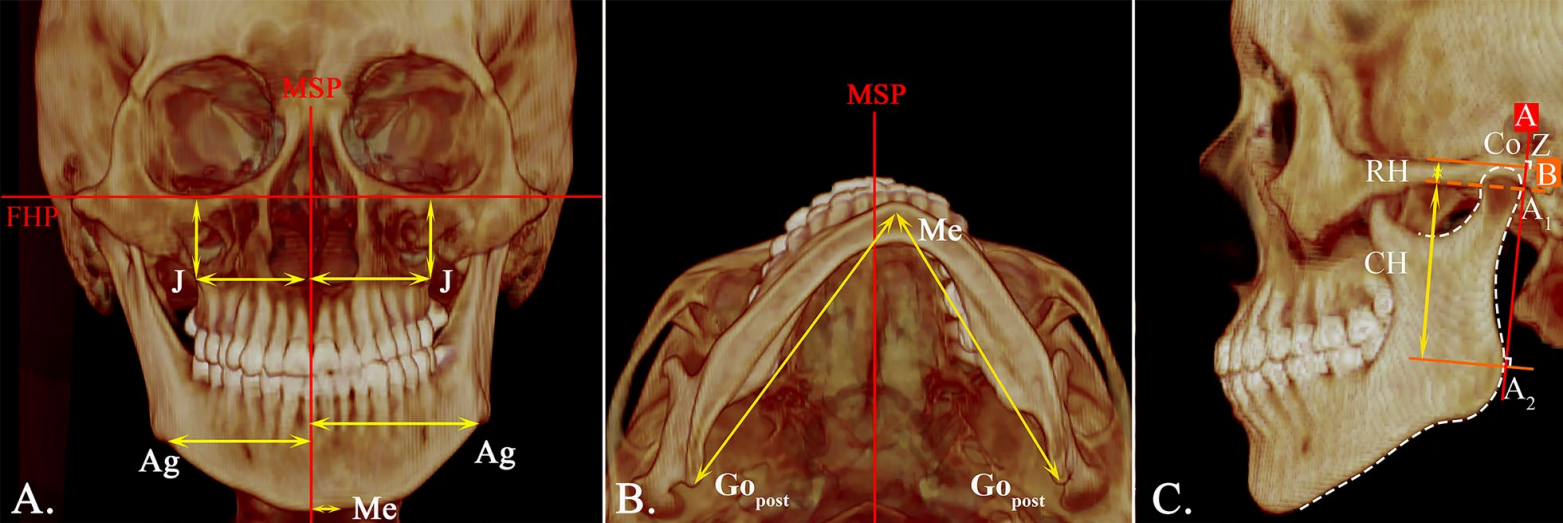
Landmarks and measurements in three-dimensional skeletal analysis. **A** Coronal view; **B** axial view; **C** sagittal view

**Fig. 2:**
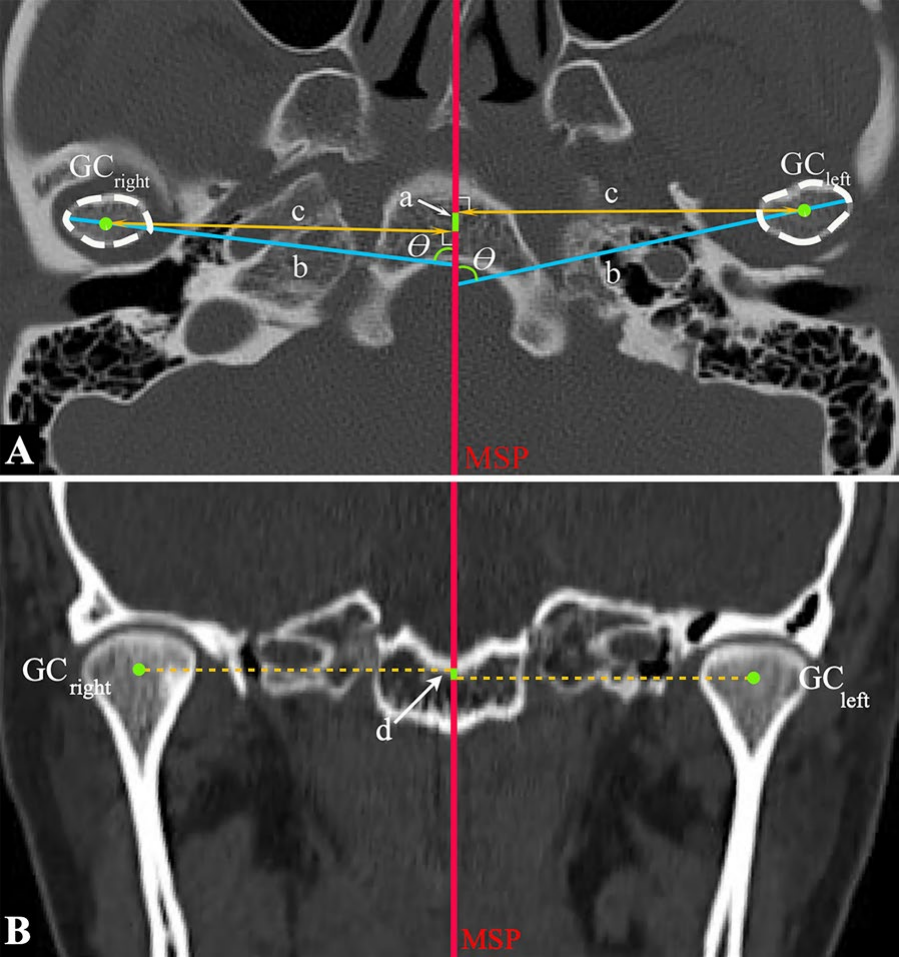
Positional assessments of the condylar. **A** Measurements of the anteroposterior relationship and lateromedial displacement of the condylar. GC, the geometric center of the condyle; a, the anteroposterior distance difference between GC of the right and left condyle to the MSP; b, the long axis of the condylar process; c, the distance between CG of the right and left condyle to the MSP; θ, the angle between the long axis of the condylar process and the MSP. **B** Measurements of the vertical relationship of the condylar. d, the vertical distance difference between GC of the right and left condyle to the MSP

**Fig. 3:**
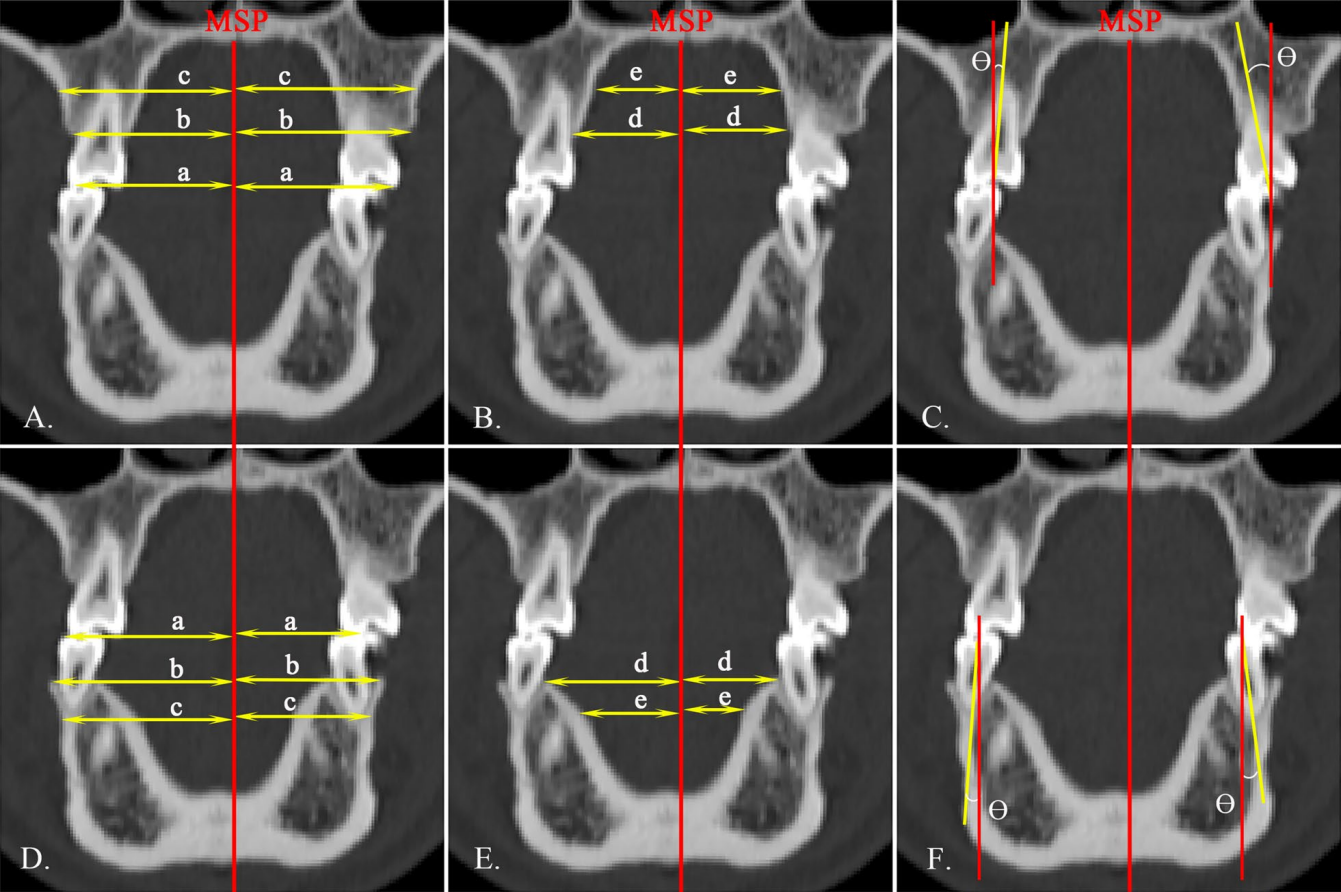
Dentoalveolar measurements of second premolars on coronal view. **A**, **D** a, the transverse width of the second premolar, distance from buccal cusp to the MSP; b, bucco alveolar crest width; c, bucco midalveolar widths; **B**, **E** d, palatal alveolar crest width; e, palatal midalveolar widths; **C**, **F** θ, the inclination of the second premolar

All subjects had CBCT scans (Philips MX 16-slice) taken in maximum intercuspation, which were obtained at 90 kV and 40 mA with a slice thickness of 0.49 mm. Digital Imaging and Communications in Medicine (DICOM) files obtained from the CBCT scans were reconstructed using Dolphin 3D (Dolphin Imaging, version 11.7, Chatsworth, CA91311, USA) software. Reorientation of each scan was performed using the standardized 3D reference planes. The landmarks, measurements, and reference planes selected for this investigation were shown in Figs. 1, 2, and 3. The following reference planes were used to ensure the consistent orientation of the 2D cross-sectional slices: (1) Frankfort horizontal (FH) plane: plane passing through right porion, left porion, and midpoint of left and right orbitale, (2) midsagittal plane (MSP): plane perpendicular to FH plane, passing through the neck of the crista galli and Opisthion, (3) coronal plane: plane perpendicular to FH plane and MSP, passing through Op.

### Skeletal analysis

On the coronal view, the jugal process (J) and antegonial (Ag) widths on each side were assessed relative to MSP. The sum of both sides represented the total width of the maxilla (J–J) and mandible (Ag–Ag) respectively. The maxillomandibular width difference was calculated by subtracting the total maxillary width with the total mandibular width [(J–J) − (Ag–Ag)] (Fig. 1A). Menton (Me) deviation was measured relative to MSP to indicate mandibular displacement. The corpus length of the mandible was measured for both sides from the most posterior point of the gonial angle, gonion posterius (Go_post_) to the menton (Me) on the axial view (Fig. 1B).

Maxillary vertical assessments were investigated by measuring the distance from the jugal process to the FH plane (J-FH) (Fig. 1A). The vertical assessments of the mandible were made according to Habets’ technique [19] and made separately for both sides on the sagittal view of the CBCT image. The most posterior points on the condyle and ramus were marked as A_1_ and A_2_, respectively. A line that passed through points A_1_ and A_2_ were termed the A-line. A second line drawn from the most superior point of the condylar (C_o_) and perpendicular to the A-line, was termed the B-line. The intersection of the A- and B-line was called point Z. The distances between points A_1_ and Z were measured as condylar height (CH). Similarly, the distances between points A_1_ and A_2_ were measured as ramus height (RH), and the distances between points Z and A_2_ were measured as condylar plus ramus heights (CH + RH) (Fig. 1C). The asymmetry index [19] of condylar height (CAI), ramal height (RAI), and condylar plus ramal height (CRAI) were calculated based on the following formula:

$${\text{Asymmetry}}\;{\text{index:}}\;[\left( {{\text{Right}} - {\text{Left}}} \right)/\left( {{\text{Right}} + {\text{Left}}} \right)] \times 100\%$$

### Condylar analysis

The geometric center (GC) of the condyles was first identified on the axial view. Then, the anteroposterior [20] (Fig. 2A) and vertical [21] (Fig. 2B) relationship of the condylar was measured by calculating the distance difference between the GC of the right and left condyle to the MSP. The right or the crossbite side of the condyle was considered as 0 point [20]. A positive value indicates that the left or the non-crossbite side of the condyle was positioned anterior or higher to the 0 point, and a negative value indicates a posterior or lower position. The transverse position of the condylar was measured by calculating the distance between the GC of the condylar and MSP. The lateromedial displacement of the condyle was assessed by measuring the angle between the long axis of the condylar process and the MSP (Fig. 2A).

### Dentoalveolar analysis

**Fig. 4:**
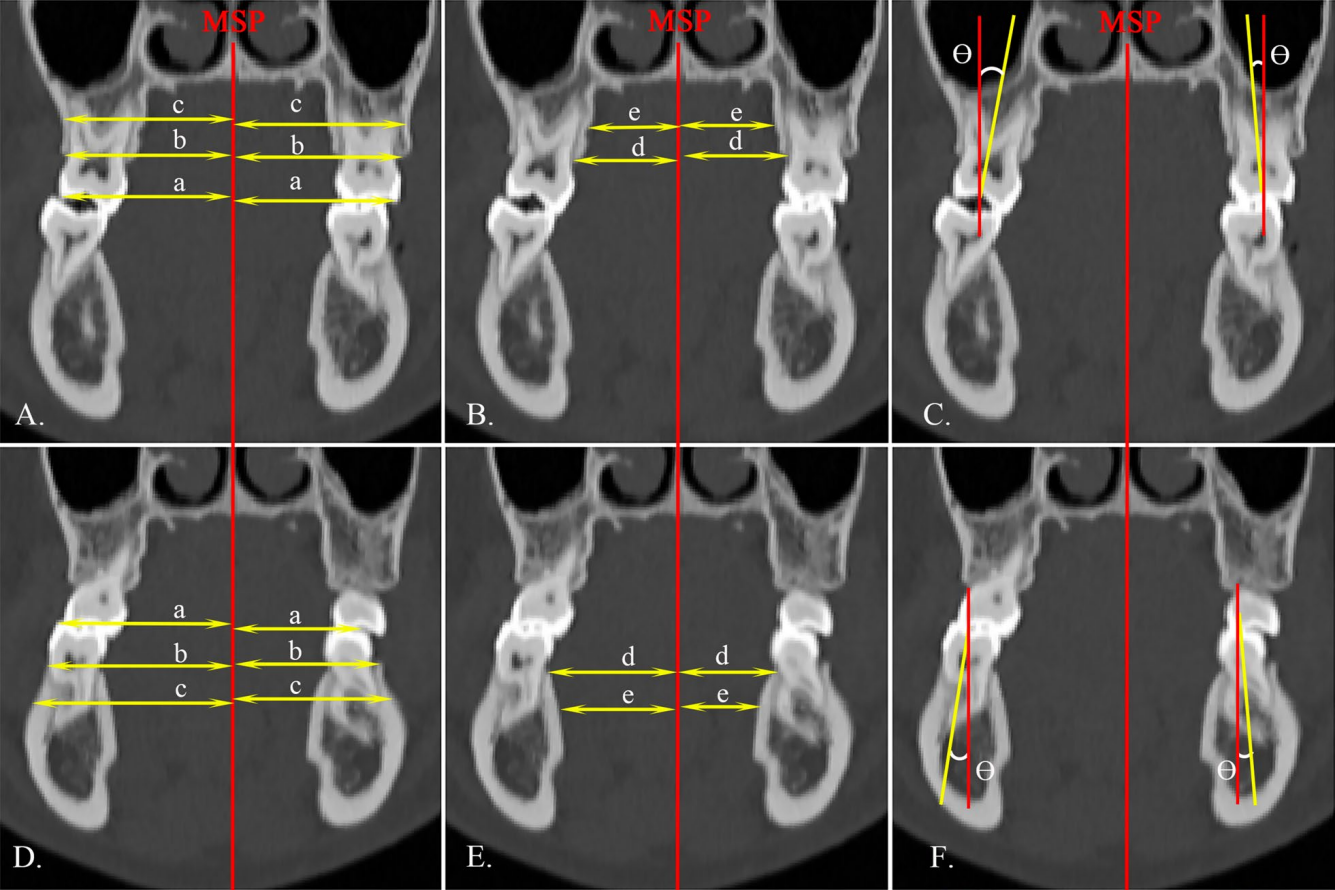
Dentoalveolar measurements of first molars on coronal view. **A**, **D** a, the transverse width of the first molar, distance from mesiobuccal cusp to the MSP; b, bucco alveolar crest width; c, bucco midalveolar widths; **B**, **E** d, palatal alveolar crest width; e, palatal midalveolar widths; **C**, **F** θ, the inclination of the first molar

Dentoalveolar measurements were made on the second premolar (Fig. 3) and first molar (Fig. 4) of both arches. The midalveolar widths of the maxilla and mandible were determined at 7 mm apical to the alveolar crest [14]. The inclination of the second premolar and the first molar was determined as the angle between the long axis of the tooth and the vertical reference line. For single-rooted molar and premolar, the long axis was defined as the line connecting the groove between the buccal and palatal cusps and the root apex. For multi-rooted molar and premolar, the long axis was defined as the line connecting the groove between the buccal and palatal cusps and the furcation of the roots.

The difference between bilateral structures was calculated by right or crossbite side minus left or non-crossbite side. For all linear measurements, a positive value indicates the right or crossbite side is larger than the left or non-crossbite side, and the opposite for the negative value. The maxillomandibular width difference was calculated by subtracting the total maxillary width from the total mandibular width. For menton deviations, a positive value indicates the mandible was displaced toward the right or the crossbite side, and a negative value indicates the mandible was displaced toward the left or the non-crossbite side. For tooth inclinations, a positive value indicates a buccoversion of the crown and a negative value indicates a linguoversion of the crown in relation to root apex (single root) or root furcation (multirooted tooth).

### Statistical analysis

A priori sample size calculation was performed with PASS Sample Size Software V.15 (NCSS LLC., 121 Kaysville, Utah, USA). Mean and standard deviation of mandibular body length on the crossbite side in UPCB adults (81.46 ± 4.95) and the right side on the normal occlusion adults (72.12 ± 11.83) reported by Veli et al. [22] was taken as the reference value. The power was set at 80%, and a statistical significance of 0.05 and an effect size of 0.5 was considered. As a result, the sample size calculated was 16 per group, and 20 samples were selected for each group.

To prevent inter-observer error, all the procedures were performed by one author. All measurements were repeated for 10 randomly selected CBCTs after at least a one-week interval to determine the measurement error. A paired *t*-test and Bland & Altman plot were applied to access the reproducibility of the measurements, and no significant differences were found between the first and second measurements. The error of the method was calculated with the intraclass correlation coefficient (ICC).

The data were determined to have a normal distribution when assessed with the Shapiro-Wilks test. Mean and standard deviation values were calculated for all normalized measurements in each of the corresponding groups. The paired *t*-test was used for the intragroup comparison whereas the intergroup comparison was analyzed using an independent *t*-test.

## Results

The reliability of all measurements was 0.923–0.989 based on the ICC test. The patients’ characteristics and demographics were summarized in Table 2. There was no gender difference between groups. Therefore, the data were pooled from male and female groups for analysis. No side-specific prevalence difference was observed in the UPCB group, thus the measurements from the left and right sides of UPCB group were combined and evaluated as crossbite and non-crossbite sides.

**Table 2:** Summary of patient characteristics and demographics

Variables	Control group (n = 20)	UPCB group (n = 20)	*P* value
*Gender*^*a*^			
M, n (%)	7 (35)	8 (40)	0.935
F, n (%)	13 (65)	12 (60)	
Age (y) (mean ± SD)^b^	27.56 ± 5.76	22.20 ± 2.88	0.000*
ANB (°) (mean ± SD)^b^	2.48 ± 1.07	1.80 ± 1.14	0.094
FMA (°) (mean ± SD)^b^	25.02 ± 3.15	26.15 ± 3.53	0.153

*UPCB* unilateral posterior crossbite; *M* male; *F* female; *n* number; *SD* standard deviation; *ANB* Angle formed by A-point-nasion-B-point; *FMA* the angle between FH plane and mandibular plane

^a^Chi-square test

^b^Independent *t*-test

Statistically significant when *(*P* < 0.05)

The Control group showed significant side differences in mandibular corpus length (*P* = 0.002) and angle of the condylar process (*P* = 0.044). In the UPCB group, RH (*P* = 0.004), CH + RH (*P* = 0.003), and mandibular corpus length (*P* = 0.000) were significantly greater on the non-crossbite side. Antegonial widths were found to be significantly higher on the crossbite side (*P* = 0.000, Table 3). Both groups showed transverse dental asymmetries in linear and angular measurements, and the difference in UPCB group was more significant (*P* < 0.05, Table 4). In UPCB group, the maxillary posterior teeth were more buccally inclined, and mandibular posterior teeth were lingually inclined on the crossbite side. Mandibular posterior teeth were more upright on the non-crossbite side (Table 4).

**Table 3:** Statistical Side Comparisons of Skeletal Variables in the Control Group and UPCB Group

	Control group				UPCB Group			
	Right side	Left side			Crossbite side	Non-crossbite side		
	Mean ± SD	Mean ± SD	*t* value	*P* value	Mean ± SD	Mean ± SD	*t* value	*P* value
*Morphological measurements of maxilla and mandible*								
Vertical								
Maxillary height (mm)	29.26 ± 2.81	29.23 ± 2.88	− 0.343	0.735	30.23 ± 2.68	30.72 ± 3.08	− 1.946	0.067
CH (mm)	7.47 ± 1.70	7.22 ± 1.84	0.416	0.682	7.28 ± 2.14	8.01 ± 1.64	− 1.102	0.284
RH (mm)	42.18 ± 3.58	41.77 ± 4.33	0.474	0.641	41.32 ± 4.63	45.50 ± 3.30	− 3.247	0.004*
CH + RH (mm)	49.64 ± 3.32	48.99 ± 4.26	0.933	0.363	48.60 ± 5.72	53.50 ± 3.09	− 3.337	0.003*
Sagittal								
Mandibular corpus length (mm)	82.24 ± 4.25	80.67 ± 4.31	3.633	0.002*	78.34 ± 5.08	81.99 ± 5.39	− 6.731	0.000*
Transverse								
Jugal process width (mm)	32.99 ± 1.95	32.66 ± 1.98	0.948	0.355	32.18 ± 2.35	31.87 ± 2.09	1.021	0.320
Antegonial width (mm)	43.31 ± 2.67	43.64 ± 3.50	− 0.378	0.710	46.60 ± 2.29	40.05 ± 3.27	7.755	0.000*
Positional measurements of condylar								
Anteroposterior difference of condylar process (mm)	0 ± 0	0.22 ± 2.09	0.470	0.644	0 ± 0	0.96 ± 3.45	− 1.241	0.230
Vertical difference of condylar process (mm)	0 ± 0	0.35 ± 1.21	− 1.30	0.209	0 ± 0	0.17 ± 2.18	− 3.40	0.737
Transverse position of condylar process (mm)	53.22	53.09	− 0.429	0.673	52.12 ± 2.76	51.98 ± 2.78	0.271	0.789
Angle of condylar process (°)	74.69 ± 6.63	72.47 ± 5.23	2.159	0.044*	73.99 ± 8.38	76.08 ± 6.50	− 0.991	0.334

*UPCB* unilateral crossbite; *SD* standard deviation; *CH* condylar height; *RH* ramal height; *CH* + *RH* condylar-plus-ramal height

Statistically significant when *(*P* < 0.05)

**Table 4:** Statistical side comparisons of dentoalveolar variables in the control group and UPCB group

	Control group				UPCB group			
	Right side	Left side			Crossbite side	Non-crossbite side		
	Mean ± SD	Mean ± SD	*t* value	*P* value	Mean ± SD	Mean ± SD	*t* value	*P* value
*Linear measurements*								
Maxillary transverse width—premolar								
Premolar (mm)	24.28 ± 2.16	25.31 ± 1.52	− 1.808	0.086	26.19 ± 2.62	23.98 ± 1.69	3.909	0.001*
Buccal alveolar crest (mm)	26.11 ± 1.92	26.35 ± 1.83	− 0.457	0.645	26.56 ± 1.66	25.43 ± 1.34	2.729	0.013*
Buccal midalveolar (mm)	26.95 ± 2.05	26.99 ± 1.59	− 0.076	0.940	27.32 ± 1.70	27.14 ± 1.88	0.505	0.620
Palatal alveolar crest (mm)	17.20 ± 2.11	17.47 ± 1.82	− 0.520	0.609	18.10 ± 2.09	16.93 ± 1.41	2.443	0.024*
Palatal midalveolar (mm)	13.67 ± 1.63	14.29 ± 1.62	− 1.223	0.236	13.64 ± 1.64	14.38 ± 2.06	− 1.725	0.101
Maxillary transverse width—molar								
Molar (mm)	27.58 ± 2.03	28.37 ± 1.63	− 1.784	0.090	29.59 ± 2.52	26.44 ± 1.34	4.955	0.000*
Buccal alveolar crest (mm)	29.05 ± 2.00	29.59 ± 1.63	− 1.317	0.204	29.32 ± 3.21	28.56 ± 1.60	0.966	0.346
Buccal midalveolar (mm)	32.02 ± 1.88	32.29 ± 1.93	− 0.689	0.499	31.47 ± 3.38	31.58 ± 1.77	− 0.155	0.878
Palatal alveolar crest (mm)	17.61 ± 1.79	18.31 ± 1.32	− 1.810	0.086	18.62 ± 2.28	17.17 ± 1.23	2.605	0.017*
Palatal midalveolar (mm)	15.11 ± 1.51	15.49 ± 1.39	− 0.882	0.389	15.77 ± 2.03	15.11 ± 1.75	1.295	0.211
Mandibular transverse width—premolar								
Premolar (mm)	20.18 ± 2.34	21.45 ± 1.40	− 2.302	0.033*	25.77 ± 3.12	18.00 ± 1.05	9.764	0.000*
Buccal alveolar crest (mm)	23.80 ± 2.11	24.57 ± 1.61	− 1.340	0.196	28.47 ± 2.08	20.26 ± 1.66	13.279	0.000*
Buccal midalveolar (mm)	24.97 ± 2.26	26.25 ± 1.89	− 1.859	0.079	29.05 ± 2.77	20.73 ± 1.72	10.687	0.000*
Lingual alveolar crest (mm)	15.98 ± 2.37	17.04 ± 1.37	− 1.677	0.110	21.03 ± 2.86	13.52 ± 2.77	6.731	0.000*
Lingual midalveolar (mm)	11.14 ± 3.18	12.31 ± 1.83	− 1.450	0.163	18.63 ± 2.72	9.18 ± 5.39	9.343	0.000*
Mandibular transverse width—molar								
Premolar (mm)	24.09 ± 1.98	25.51 ± 1.30	− 3.380	0.003*	29.07 ± 2.05	22.65 ± 1.65	12.467	0.000*
Buccal alveolar crest (mm)	28.01 ± 1.94	29.18 ± 1.51	− 2.437	0.025*	32.70 ± 2.06	24.981 ± 1.52	13.440	0.000*
Buccal midalveolar (mm)	30.65 ± 2.24	32.24 ± 1.67	− 2.586	0.018*	34.89 ± 2.22	26.45 ± 1.66	11.895	0.000*
Lingual alveolar crest (mm)	18.30 ± 2.17	19.55 ± 1.43	− 2.446	0.024*	23.71 ± 1.81	15.31 ± 1.37	16.887	0.000*
Lingual midalveolar (mm)	15.99 ± 2.36	17.00 ± 1.68	− 1.717	0.102	21.99 ± 2.65	12.23 ± 1.84	15.682	0.000*
*Angular measurements*								
Inclinations if maxillary teeth								
Premolar (°)	0.67 ± 5.22	1.52 ± 3.29	− 0.740	0.468	3.90 ± 6.82	− 1.43 ± 4.36	3.198	0.005*
Molar (°)	3.90 ± 5.04	7.63 ± 3.61	− 3.409	0.003*	9.25 ± 8.38	0.14 ± 3.90	4.104	0.001*
Inclinations if mandibular teeth								
Premolar (°)	− 9.08 ± 5.22	− 10.64 ± 4.03	1.198	0.246	− 13.91 ± 5.35	− 3.85 ± 4.75	− 6.519	0.000*
Molar (°)	− 15.47 ± 3.55	− 15.95 ± 4.11	0.323	0.751	− 17.45 ± 6.20	− 9.27 ± 5.09	− 4.542	0.000*

*SD* standard deviation; *UPCB* unilateral posterior crossbite

Statistically significant when *(*P* < 0.05)

Maxillomandibular skeletal vertical asymmetry and condylar asymmetry were not statistically significant between groups (*P* > 0.05). Although the UPCB group showed a significantly larger antegonial width difference (*P* < 0.05), no significant difference was seen in the interantegonial width and maxillomandibular width difference (*P* > 0.05) when compared to the control group. The differences in mandibular corpus length and menton displacement were significantly greater in UPCB group than in control group (*P* < 0.05, Table 5). The difference in mandibular dentoalveolar width measurements was significantly greater in UPCB group compared to control group (*P* < 0.05). However, in the maxilla, significant differences were only observed in the maxillary premolar width, maxillary molar width, and maxillary molar palatal alveolar crest width in comparison to control group. The changes in the buccolingual inclination of posterior teeth in both arches were greater in UPCB group (*P* < 0.05, Table 6).

**Table 5:** Inter-group comparisons of skeletal variables between the control group and UPCB group

	Control group		UPCB group			
	Mean ± SD	95% CI	Mean ± SD	95% CI	*t* value	*P* value
*Morphological measurements of the maxilla and mandible*						
Vertical						
Maxillary height difference (mm)	0.34 ± 0.44	− 0.17, 0.24	− 0.49 ± 1.13	− 1.02, 0.03	1.94	0.064
CAI (%)	14.41 ± 10.08	9.69, 19.13	17.9 ± 11.49	12.53, 23.29	− 1.023	0.313
RAI (%)	3.53 ± 3.03	2.09, 4.93	6.20 ± 5.85	3.46, 8.94	− 1.822	0.079
CRAI (%)	2.59 ± 1.99	1.65, 3.52	7.92 ± 11.75	2.43, 13.43	− 2.00	0.059
Sagittal						
Mandibular corpus length difference (mm)	1.58 ± 1.94	0.67, 2.48	− 3.65 ± 2.42	− 4.79, − 2.52	7.53	0.000*
Transverse						
Jugal process width difference (mm)	0.93 ± 3.01	− 0.48, 2.34	0.18 ± 1.12	− 0.35, 0.70	0.426	0.672
Antegonial width difference (mm)	− 0.33 ± 3.91	− 2.16, 1.50	6.54 ± 3.77	4.78, 8.31	− 5.659	0.000*
Interjugular width (mm)	65.65 ± 3.61	63.96, 67.33	65.14 ± 3.96	65.14, 66.99	1.051	0.304
Interantegonial width (mm)	86.66 ± 4.90	84.68, 89.22	86.65 ± 4.21	84.68, 88.61	0.212	0.833
Maxillomandibular width difference (mm)	− 21.31 ± 3.85	− 23.11, − 19.50	− 22.60 ± 3.89	− 22.41, − 20.78	1.058	0.297
Menton deviation (mm)	− 0.49 ± 2.54	− 1.67, 0.70	3.18 ± 5.01	0.83, 5.53	− 2.922	0.006*
Positional measurements of the condylar						
Difference of condylar process anteroposterior position (mm)	0.22 ± 2.09	− 0.76, 1.20	0.96 ± 3.46	− 0.66, 2.58	− 0.819	0.418
Difference of condylar process vertical position (mm)	0.44 ± 1.25	− 0.14,1.02	0.17 ± 2.17	− 0.85, 1.18	0.491	0.626
Difference of condylar process transverse position (mm)	0.13 ± 1.35	− 0.50, 0.76	0.13 ± 2.20	− 0.90, 1.16	− 0.006	0.995
Difference of condylar process angle (°)	2.22 ± 4.59	0.07, 4.36	− 2.09 ± 9.43	− 6.50, 2.32	1.836	0.077

*UPCB* unilateral posterior crossbite; *SD* standard deviation; *CI* confidence interval; *CAI* condylar asymmetry index; *RAI* ramal asymmetry index; *CRAI* condylar-plus-ramal asymmetry index

Statistically significant when *(*P* < 0.05)

**Table 6:** Inter-group comparisons of Dentoalveolar variables between the control group and UPCB group

	Control group		UPCB group			
	Mean ± SD	95% CI	Mean ± SD	95% CI	*t* value	*P* value
*Linear measurements*						
Maxillary transverse width differences—premolar						
Premolar (mm)	− 1.03 ± 2.55	− 1.10, − 2.22	2.21 ± 2.53	1.03, 3.40	− 4.307	0.000*
Buccal alveolar crest (mm)	− 0.25 ± 2.40	− 1.37, 0.88	1.13 ± 1.84	0.26, 1.99	− 2.026	0.050
Buccal midalveolar (mm)	− 0.04 ± 2.07	− 1.00, 0.93	0.18 ± 1.59	− 0.57, 0.93	− 0.368	0.715
Palatal alveolar crest (mm)	− 0.28 ± 2.36	− 1.38, 0.83	1.17 ± 2.13	0.17, 2.16	− 2.023	0.050
Palatal midalveolar (mm)	− 0.62 ± 2.25	− 1.67, 0.44	− 0.74 ± 1.92	− 1.64, 0.16	0.189	0.851
Maxillary transverse width differences—molar						
Molar (mm)	− 0.80 ± 1.99	− 1.73, 0.14	3.16 ± 2.85	1.82, 4.49	− 5.082	0.000*
Buccal alveolar crest (mm)	− 0.55 ± 1.85	− 1.41, 1.85	0.76 ± 3.52	− 0.89, 2.41	− 1.467	0.151
Buccal midalveolar (mm)	− 0.27 ± 1.75	− 1.09, 0.55	− 0.11 ± 3.02	− 1.52, 1.31	− 0.211	0.834
Palatal alveolar crest (mm)	− 0.70 ± 1.72	− 1.50, 0.11	1.45 ± 2.48	0.28, 2.48	− 3.172	0.003*
Palatal midalveolar (mm)	− 0.38 ± 1.90	− 1.27, 0.52	0.66 ± 2.28	− 0.41, 1.73	− 1.559	0.127
Mandibular transverse width differences—premolar						
Premolar (mm)	− 1.27 ± 2.47	− 2.43, − 0.12	7.77 ± 3.56	6.10, 9.43	− 9.334	0.000*
Buccal alveolar crest (mm)	− 0.77 ± 2.55	− 1.96, 0.43	8.22 ± 2.77	6.92, 9.51	− 10.667	0.000*
Buccal midalveolar (mm)	− 1.28 ± 3.07	− 2.71, 0.16	8.32 ± 3.48	6.69, 9.95	− 9.247	0.000*
Lingual alveolar crest (mm)	− 1.06 ± 2.81	− 2.37, 0.26	7.51 ± 4.99	5.17, 9.85	− 6.687	0.000*
Lingual midalveolar (mm)	− 1.18 ± 3.62	− 2.87, 0.52	9.46 ± 4.53	7.34, 11.57	− 8.198	0.000*
Mandibular transverse width differences—molar						
Molar (mm)	− 1.43 ± 1.89	− 2.31, − 0.54	6.42 ± 2.30	5.34, 7.49	− 11.785	0.000*
Buccal alveolar crest (mm)	− 1.17 ± 2.15	− 2.17, − 0.17	7.72 ± 2.57	6.52, 8.92	− 11.876	0.000*
Buccal midalveolar (mm)	− 1.59 ± 2.28	− 2.87, − 0.30	8.44 ± 3.17	6.95, 9.92	− 10.691	0.000*
Lingual alveolar crest (mm)	− 1.25 ± 2.28	− 2.31, − 0.18	8.40 ± 2.23	7.36, 9.44	− 13.553	0.000*
Lingual midalveolar (mm)	− 1.02 ± 2.64	− 2.25, 0.22	9.76 ± 2.78	8.46, 11.06	− 12.554	0.000*
*Angular measurements*						
Maxillary teeth inclination differences (°)						
Premolar (°)	− 1.12 ± 4.39	− 3.17, 0.94	5.37 ± 7.35	1.93, 8.80	− 3.385	0.002*
Molar (°)	− 3.74 ± 4.90	− 6.03, − 1.44	9.10 ± 9.92	4.46, 13.75	− 5.189	0.000*
Mandibular teeth inclination differences (°)						
Premolar (°)	1.57 ± 5.84	− 1.17, 4.30	− 10.06 ± 6.90	− 13.28, − 6.83	5.748	0.000*
Molar (°)	0.48 ± 6.66	− 2.63, 3.59	− 8.18 ± 8.05	− 11.94, − 4.41	3.707	0.001*

*UPCB* unilateral posterior crossbite; *SD* standard deviation; *CI* confidence interval

Statistically significant when *(*P* < 0.05)

## Discussion

We measured the skeletal and dentoalveolar variables of subjects with normal occlusion and growth to establish baselines so that the amount of modification in skeletal Class I UPCB adults can be compared. Young adults aged between 18 and 40 were included in the present study to avoid the influence of potential growth. To exclude UPCB resulting from unilateral condylar hyperplasia, only skeletal Class I adults with continuous condylar cortical bone were included in the present study [23]. We do not specifically include the examination of functional shift into the sample selection criteria because, (1) as mentioned earlier, the functional shift is hardly detected in adults with UPCB due to progressive musculoskeletal adaptation; (2) we were not able to carry out physical examinations on each adult patient because the CBCT scans obtained were not limited to orthodontic patients only.

It had been reported that facial asymmetry is a naturally occurring phenomenon [24]. The present results showed minor asymmetries in the control group, dentoskeletal asymmetries with linear changes were observed at most 4 mm, and angular differences of at most 2°. Consistent with Veli et al. [22], our result suggested that in skeletal side comparisons, only mandibular corpus length received the statistically significant difference in the control group. These suggested that the transverse dental asymmetry in the control group may be the result of camouflaging skeletal deficiency, to maintain a Class I relationship. Patients with no perceivable facial asymmetry were included in the current study as a control group. Thus, we attribute these differences to the asymmetry in nature.

We observed UPCB adults with normal maxillary (J-J) and mandibular (Ag–Ag) skeletal widths and showed more asymmetry in the mandible compared to the control group, mainly in the measurement of the antegonial width (Ag-MSP) and the corpus length. We indicate the increase in antegonial width (Ag-MSP) on the crossbite side was due to the difference in mandibular corpus length. Alongside the corpus length difference, menton was shown to be displaced toward the side of the shorter corpus length. Since the critical distance of menton deviations that differentiates facial symmetry from asymmetry is approximately 4 mm [25, 26], we considered the subjects in the control group (− 0.49 ± 2.54 mm) to be symmetric but not for the UPCB group (3.18 ± 5.01 mm) due to large standard deviations.

The RH and CH + RH values were significantly shorter on the crossbite side than on the non-crossbite side in UPCB group while there was no side difference in control group. The decrease in RH and CH + RH on the crossbite side was consistent with the studies of kilic [28]. These differences might be because of the restriction in mandibular growth due to forced occlusion [5], adaptive chewing pattern [8], or impaired functional activity of the masticatory muscles [27]. According to Habets et al. [19], the asymmetric index value greater than 3% could be considered mandibular vertical asymmetry. In our study, apart from the CRAI in the control group, both control and UPCB groups’ asymmetric indexes were higher than the 3% threshold, and intergroup comparisons were not statistically significant. Some authors [10, 28] explained that the high CAI values as a result of mandibular shift due to occlusal change and TMJ remodeling, and the differences in the measurement methods might also be a reason. Our study was compared with Halicioglu et al [29], as they used the same radiographic assessment as we did. Consistent with our results, they found no statistically significant differences in CAI, RAI, and CRAI values among the control and UPCB groups.

Additionally, assessments of condylar positional asymmetries showed no significant differences in both groups. We inferred these negative findings to the variability of condylar position based on the large standard deviation observed. Few studies [30, 31] investigated the true skeletal asymmetry and found that condylar asymmetry was influenced by the condylar morphology, coronoid process, and the glenoid fossa. Changes in dimension or bony deposition on these structures were a sign of adaptation to form a better occlusion.

Corroborating with the previous belief [7], the present study showed that UPCB adults displayed more transverse asymmetry than normal occlusion adults on dentoalveolar variables, predominately in the mandible. The increases in widths of maxillary premolar, molar, and palatal alveolar crest were statistically significant and were consistent with the greater buccal inclinations of the respective maxillary teeth on the crossbite side. In the mandible, an increase in the alveolar width was observed on the crossbite side with lingually inclined posterior teeth. We explained these phenomena as a compensation of the alveolar bone to the degree of malocclusion or vice versa. Also, significant transverse deviation in UPCB group on the mandibular posterior region might be due to a mandibular functional shift, dentoalveolar or skeletal asymmetry, or a combination of these factors. As the present study observed no condylar positional asymmetry, the mandibular displacement caused by asymmetric corpus length shown in our results might contribute to the unilateral posterior crossbite in adult patients, and the posterior teeth on both arches showed compensations to overcome these skeletal discrepancies. Our findings provide some guidance in the clinical practice (1) improvement of facial asymmetry is difficult to achieve simply by orthodontic treatment in UPCB adults; (2) asymmetry treatment modalities, such as asymmetric maxillary expansion, should be considered for the treatment of adults with UPCB, especially when maxillary posterior teeth on both sides displayed compensations in opposite direction; (3) early intervention of UPCB is crucial for the prevention of skeletal asymmetry.

The limitations of this study are the small sample size and deficiency in the assessment of potential functional shift. Most of the variables measured in this study appeared with a relatively large 95% CI, we contributed this phenomenon to the limitation of sample size and the complexity of multiple anatomical interactions of craniofacial structures. The data collection and measurements were made by one author, and all the variables measured in crossbite groups were compared to the control group to reduce variability to overcome the limitation in the sample size. The present study focused on the three-dimensional linear and angular measurements of maxillomandibular skeletal and transverse dentoalveolar asymmetries in UPCB adults. To further investigate their dentoskeletal adaption patterns, alveolar bone height together with volumetric and surface measurements of the TMJ complex and other craniofacial structures can also be performed in future studies.

## Conclusions

1. Adults with UPCB showed significant asymmetry in dentoskeletal measurements in the transverse dimension.
2. No significant asymmetry was found in the condylar position and the maxillomandibular height in UPCB group when compared to the control group.
3. Mandibular corpus length asymmetry and menton deviation were observed in both groups, with UPCB adults showing greater deviations.
4. In UPCB group, the maxillary posterior teeth were more buccally inclined, and mandibular posterior teeth were lingually inclined on the crossbite side. Mandibular posterior teeth were more upright to accommodate the lingually inclined maxillary posterior teeth on the non-crossbite side.

## Data Availability

The datasets used and/or analyzed during the current study are available from the corresponding authors upon reasonable request.

## Abbreviations

UPCB: Unilateral posterior crossbite
CBCT: Cone-beam computed tomography
UPCB: Unilateral posterior crossbite
2D: Two-dimensional
3D: Three-dimensional
DICOM: Digital Imaging and Communications in Medicine
FH plane: Frankfort horizontal plane
MSP: Midsagittal plane
Op: Opisthion
J: Jugal process
Ag: Antegonial
J–J: Total width of the maxilla
Ag–Ag: Total width of the mandible
(J-J) − (Ag–Ag): Maxillomandibular width difference
Me: Menton
Go_post_: Gonion posterius
CH: Condylar height
RH: Ramal height
CH + RH: Condylar plus ramal height
CAI: Condylar asymmetry index
RAI: Ramal asymmetry index
CRAI: Condylar-plus-ramal asymmetry index
GC: Geometric center
ICC: Intraclass correlation coefficient
